# Extracellular Microvesicle MicroRNAs and Imaging Metrics Improve the Detection of Aggressive Prostate Cancer: A Pilot Study

**DOI:** 10.3390/cancers17050835

**Published:** 2025-02-27

**Authors:** Kapil K. Avasthi, Jung W. Choi, Tetiana Glushko, Brandon J. Manley, Alice Yu, Jong Y. Park, Joel S. Brown, Julio Pow-Sang, Robert Gantenby, Liang Wang, Yoganand Balagurunathan

**Affiliations:** 1 Department of Tumor Microenvironment and Metastasis, Moffitt Cancer Center, Tampa, FL 33612, USA; kapil.avasthi@moffitt.org; 2 Department of Diagnostic & Interventional Radiology, Moffitt Cancer Center, Tampa, FL 33612, USA; jung.choi@moffitt.org (J.W.C.); robert.ganteby@moffitt.org (R.G.); 3 Department of Genitourinary Oncology, Moffitt Cancer Center, Tampa, FL 33612, USA; brandon.manley@moffitt.org (B.J.M.); alice.yu@moffitt.org (A.Y.); julio.powsang@moffitt.org (J.P.-S.); 4 Department of Cancer Epidemiology, Moffitt Cancer Center, Tampa, FL 33612, USA; jong.park@moffitt.org; 5 Department of Mathematical Oncology, Moffitt Cancer Center, Tampa, FL 33612, USA; joel.brown@moffitt.org; 6 Department of Machine Learning, Moffitt Cancer Center, Tampa, FL 33612, USA

**Keywords:** liquid biopsy, miRNA and imaging biomarkers, prostate cancer, radiomics of MRI

## Abstract

**Background/Objectives:** Prostate cancer (PCa) is the most diagnosed cancer in men worldwide. Early diagnosis of the disease provides better treatment options for these patients. Recent studies have demonstrated that plasma-based extracellular vesicle microRNAs (miRNAs) are functionally linked to cancer progression, metastasis, and aggressiveness. The use of magnetic resonance imaging (MRI) as the standard of care provides an overall assessment of prostate disease. Quantitative metrics (radiomics) from the MRI provide a better evaluation of the tumor and have been shown to improve disease detection. **Methods:** We conducted a study on prostate cancer patients, analyzing baseline blood plasma and MRI data. Exosomes were isolated from blood plasma samples to quantify miRNAs, while MRI scans provided detailed tumor morphology. Radiomics features from MRI and miRNA expression data were integrated to develop predictive models, which were evaluated using ROC curve analysis, highlighting the multivariable model’s effectiveness. **Results:** Our findings indicate that the univariate feature-based model with the highest Youden’s index achieved average areas under the receiver operating characteristic (ROC) curve of 0.76, 0.82, and 0.84 for miRNA, MR-T2W, and MR-ADC features, respectively, in identifying clinically aggressive (Gleason grade) disease. The multivariable feature-based model yielded an average area under the curve (AUC) of 0.88 and 0.95 using combinations of miRNA markers with imaging features in MR-ADC and MR-T2W, respectively. **Conclusions:** Our study demonstrates that combining miRNA markers with MRI-based radiomics improves the identification of clinically aggressive prostate cancer.

## Simple Summary

Prostate cancer is the most common cancer in men, and early detection following treatment strategies is the key factor for improved disease outcomes. This study proposes to improve the detection of aggressive disease by using multimodal predictors based on MR radiomics (imaging metrics) along with blood-based microRNA markers. We believe molecular assessment (miRNA), along with the physiology of the tumor (radiomics), provides a comprehensive assessment of the disease condition. Our proposed approach shows improved diagnoses and personalized treatments, improving patient outcomes. This multimodal approach will provide improved disease monitoring and reduce the need for invasive biopsies. Secondary validation would benefit faster clinical use.

## 1. Introduction

Prostate cancer (PCa) is the second most common cause of cancer mortality among men worldwide and represents a significant health burden [1]. PCa is a heterogeneous and multifocal disease that can appear as either a low-risk, indolent tumor confined to the prostate or a high-risk, aggressive tumor that may metastasize and become fatal if left untreated. Studies estimates that 42–66% of patients present with the indolent form of PCa [2]. The widespread adoption of serum-based prostate-specific antigen (PSA) tests has significantly improved early detection of PCa [3]. However, the PSA test lacks specificity, which has led to a higher rate of the false detection of tumors [4,5]. Most localized PCa patients with higher Gleason grades tend to be treated with radical prostatectomy (RP) as a curative option at some stage of the disease trajectory. In some of these patients, the disease progresses to present as biochemical recurrence (BCR) with an increased risk of metastasis [6,7,8,9]. There is a need to improve patient outcomes through better tailored treatment options for patients that otherwise would be over- or under-treated. This requires reliable biomarkers capable of early detection of clinically significant diseases, which can diagnose patients at a high risk of the disease and distinguish disease progression [10].

Due to its ability to assess the whole prostate gland, magnetic resonance imaging (MRI) has been adopted as the primary modality to clinically stage prostate disease [11]. The prostate imaging reporting and data system (PIRADS) allows radiological assessment of the disease. While PIRADS has improved standardized reporting, it still suffers from inter-reader variability [12]. Radiomics has developed into a methodology for characterizing tumor morphology. It provides quantitative metrics for predicting disease progression across diverse types of cancer [13,14,15].

Extracellular vascular microRNAs (miRNAs) are short, non-coding RNA molecules that regulate gene expression post-transcriptionally [16]. They play critical roles in various cellular processes and are involved in the pathogenesis of numerous diseases, including cancer [16,17]. miRNAs are typically found inside cells. However, some are shed into the circulatory system as lipid-coated particles known as exosomes [18]. Circulatory exosomal miRNAs have been identified as possible disease biomarkers as they remain relatively stable in the blood, and they are protected from endogenous RNase activity. Recently, several miRNAs have been implicated as crucial regulators in PCa progression, some targeting oncogenes that impact cancer cells’ proliferation [19,20,21,22]. These miRNAs have been shown to target the most common oncogene pathways, like the mTOR pathway [23] and cell cycle regulation [24,25]. These findings underscore the diverse roles of miRNAs in PCa pathogenesis and therapeutic responses.

In this study, we obtained morphological characterization (radiomics) of abnormal regions from PCa patients baseline bi-parametric MRI. From these patients, we also analyzed exosomal miRNAs in blood plasma. In combination, we show that these non-invasive complementary assessments (imaging and miRNAs) provide a superior patient-specific prognosis of the state of their clinically significant prostate disease. We also outline the role of multi-omic features using bi-parametric MRI (MR-T2w, MR-ADC) features and blood plasma (miRNAs) to improve predictability and allow better reproducibility across patients.

## 2. Material and Methods

### 2.1. Patient Cohort and Plasma Sample Preparation

A cohort of 48 prostate cancer patients was retrospectively obtained from The Moffitt Cancer Center. Patients who enroll in the institutional research protocol (Total Cancer Care) waive additional informed consent for an institutionally approved research study. Our retrospective research protocol was approved by the Moffitt Cancer Center/University of South Florida’s Institutional Review Board (IRB). We obtained patient baseline diagnostic multiparametric magnetic resonance imaging (MRI) before any treatment or tumor tissue biopsy. We selected patients who had prostate protocol MRIs for this study to obtain the best characterization of the gland region. The patients’ clinical record and the pathological assessment (Gleason grade) of the tumor tissue biopsy specimen were obtained from the medical record. The biopsy with the highest Gleason grade was located on the imaging scan, and the patient was assigned a pathological grade. We abstracted available clinical variables from the path records including PSA (prostate specific antigen), age, and PIRADS (prostate imaging reporting and data system).

We obtained the patients’ blood specimen (5–10 mL) that had been previously collected and stored in EDTA K2 vacutainers as part of the standard of care. For our study, each patient’s plasma sample was processed by initial centrifugation at 1300× *g* for 10 min at room temperature (RT). The resulting plasma was then transferred to a fresh 1.5 mL centrifuge tube and subjected to a second centrifugation step at 5000× *g* for 10 min at RT to obtain platelet-poor plasma. Aliquots of 250 µL from the processed plasma were quickly preserved at −80 °C until subsequent processing for exosome isolation. We formed patient sub-cohorts with the following groups: MRI (*n* = 18), blood/plasma (*n* = 48), matched imaging, and blood plasma (*n* = 13). We analyzed the multimodal data (biMRI, miRNA) cohort in all possible ways to identify discriminant features: miRNA, biMRI (T2W, ADC), and combined (miRNA and biMRI).

### 2.2. Prostate Lesion Delineation and Imaging Quantification

Multiparametric MRI was assessed by clinical radiologists (JC, TGK) who identified the most aggressive disease regions and glandular boundaries using T2-weighted (T2W) and diffusion-weighted imaging (DWI)/apparent diffusion coefficient (ADC) modalities. Abnormal regions were digitally recorded in RT (radiotherapy) format and stored in our research PACS (MIM Software^®^, Beachwood, OH, USA). Bi-parametric MRI modalities were semi-automatically registered to T2W using intensity-based image registration in Matlab^®^ (Mathworks, Natick, MA, USA, Version: 9.13.0), ensuring consistent resolution. Radiomics features (306 features spanning size, shape, and texture), compliant with Image Biomarkers Standardization Initiative (IBSI) recommendations, were extracted from abnormal regions using institutional tools for quantifying prostate abnormalities on T2W and ADC imaging [26]. The baseline MRI data were collected before PCa treatment following the institutional imaging protocol for optimal prostate characterization. Details on the radiomics descriptors used in the study are presented in Supplementary Tables S3 and S4.

### 2.3. Plasma Exosome Isolation

The exosome isolation process began with the thawing of 250 µL plasma aliquots at room temperature (RT), followed by their transfer to 1.5 mL microcentrifuge tubes. These tubes were then centrifugated at 10,000× *g* for 15 min at 4 °C to eliminate large vesicles and cellular debris, yielding a supernatant utilized for exosome isolation. The SBI SmartSEC^TM^ Single for EV Isolation^TM^ (System Biosciences, Palo Alto, CA, USA; cat# SSEC200A-1) was employed as a size exclusion chromatography-based approach. To characterize the size of the isolated particles, we utilized NanoSight NS300 with NTA Version 3.4 (build 3.4.003), as described in our previously published work [27]. The exosomes were eluted using PBS and stored at −80 °C until miRNA extraction. The plasma samples were randomized before processing, which will mitigate the batch effect in subsequent analyses.

#### 2.3.1. miRNA Extraction

The extraction of exosome miRNA was conducted using the miRNeasy (Micro) Kit (Qiagen, Valencia, CA, USA, cat# 217084). Initially, 200 µL of an exosome sample was mixed with 1 mL of QIAzol Lysis Reagent, followed by chloroform addition and centrifugation at 12,000× *g* for 15 min at 4 °C to isolate the RNA-containing aqueous phase. The extracted RNA underwent purification using the RNeasy Mini Elute spin column, involving ethanol washes and specific buffers, and elution with 15 µL of RNase-free water. The concentration of RNA was measured using the QuantiFluor^®^ RNA System (Promega, Madison, WI, USA, Cat#E3310) with Quantus equipment, and the eluted RNA was subsequently stored at −80 °C.

#### 2.3.2. Library Preparation

miRNA libraries were generated employing the QIAseq miRNA library kit (Qiagen, Valencia, CA, USA cat#331502) with 5 µL of total RNA utilized for library preparation. The process involved initial ligation of 3′ and 5′ adapters to the miRNAs. Complementary DNA (cDNA) libraries were then constructed via reverse transcription, followed by 22 cycles of PCR amplification and subsequent cleanup of cDNA using QMN beads. The concentration of the prepared libraries was quantified using the Qubit 2.0 Fluorometer with the Qubit™ dsDNA Quantification Assay Kits (Thermo Fisher Scientific, Middletown, VA, USA, cat#Q32851). Additionally, library quality was assessed using the Agilent High Sensitivity DS1000 method and the Agilent 2200 TapeStation (Agilent, Santa Clara, CA, USA, cat#5067-5585). Subsequently, libraries were pooled in an equimolar ratio based on their molarity, and the weight-to-moles conversion ratio for nucleic acids was determined.

#### 2.3.3. miRNA-Seq and Data Analysis

The miRNA-seq procedure began with pooling 20 to 24 libraries, following the guidelines outlined in the NextSeq System—Denature and Dilute Libraries Guide. To ensure quality control, 1% PhiX Control v3 was incorporated into all pools as an internal standard. Single-end read sequencing was performed with a 75 bp read length using the NextSeq 500 Sequencing System and the NextSeq 500/550 High Output v2.5 kit (75 cycles) (Illumina, San Diego, CA, USA cat#20024906).

Prior to alignment, the sequencing data’s quality control (QC) was executed using FastQC (version 0.11.9). Subsequently, adaptor removal was carried out using cutadapt (version 3.3). The adapter trimmed small RNA sequencing reads were then mapped against the miRBase database (version 21) utilizing the DNAStar tool (version 3.2). All statistical analyses were conducted within the R environment (version R4.0.3).

#### 2.3.4. Biological Pathway Enrichment Related to miRNAs

Regulatory targets and functional annotations of microRNAs were identified using TargetScan v7.2 [28] and miRDB v6.0 [29]. The Database for Annotation Visualization, and Integrated Discovery (DAVID V 6.7) was used to identify functional biological pathways for top miRNAs identified by our analysis. Furthermore, miRandav3.3a software was utilized for target prediction of the putative novel microRNA sequences [30,31].

### 2.4. Redundancy Reduction and Statistical Methods

The coefficient of determination (R^2^) between the features was computed to quantify correlation across the patient samples in our cohort. The metric (R^2^) was iteratively computed between all possible features, and highly dependent features (R^2^ ≥ 0.99) were flagged. In this dependent group, a representative feature with the highest variability across the patient population was selected, and others were removed. This process was repeated across each sub-cohort and modalities (miRNA, MR-T2W, MR-ADC) [32]. The process allowed forming a feature set that was uncorrelated (see Table 1). The level of dependency threshold needs to be balanced between removing correlated features and leaving behind those most informative. Quantitative image features used to describe the lesion of interest, computed independently in each imaging modality, are described in the Supplemental Section (see Supplementary Tables S1–S3).

**Figure 1:**
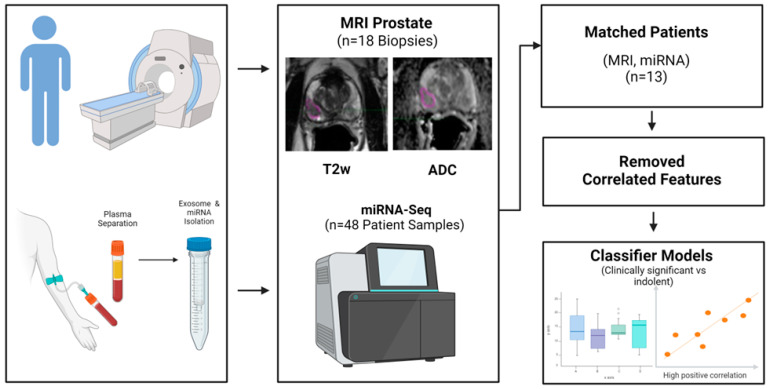
Analytical workflow to discriminate aggressive grade prostate cancer, using plasma-based miRNA and MR image based radiomics (T2W, ADC) analysis.

A logistic regression-based classifier model in univariate and multivariable (up to three dimensions) functions was then built using uncorrelated features that were identified in our cohorts. All possible combinations of features were evaluated to find the best feature combination in each cohort, which was repeated independently across the modalities. In our study, over 4.45 million possible pairs in the miRNAs cohort, over 708 thousand pairs in the MR-T2W, and 971 thousand in the MR-ADC cohort were evaluated, respectively. The feature pair was sorted based on classifier accuracy, and estimates were ensembled over random repeats. Combined multimodal features were then formed by selecting the top candidates from respective lower dimensional combinations (1, 2, and 3 pairs) in the respective modalities. The sensitivity, specificity, positive predictive value, negative predictive value, and area under receiver operator characteristics were estimated. The feature-based models were ranked based on Youden’s index (Sensitivity + Specificity − 1) and receiver operator characteristics area under the curve (ROC AUC or AUC) [33]. A hold-out cross-validation approach (80% train, 20% test) was used to estimate the model performance, which was averaged over multiple repeats (over 200). Here, we report the ensemble test statistics. Figure 1 depicts the project workflow.

## 3. Results

### 3.1. Patient Characteristics

The study included primary PCa patients with pretreatment blood plasma samples (*n* = 48) and MRI using a mixed protocol (pelvic, prostate, abdomen). Of the samples, we converged on 13 patients (18 biopsies) who had prostate MRI that followed standardized prostate imaging protocol (see Table 1). We assessed patients imaging in each of the bi-parametric modalities (miRNA, MRI-T2/ADC) that were matched with plasma-based markers to create sub-cohorts (miRNA with MR-T2W and miRNA with MR-ADC). We carried out statistical analysis to identify features that discriminate clinically significant PCa, defined by Gleason scores (GS ≥ 3 + 4), across these subgroups, considering them independently.

### 3.2. Modality Based Predictors

**Figure 2:**
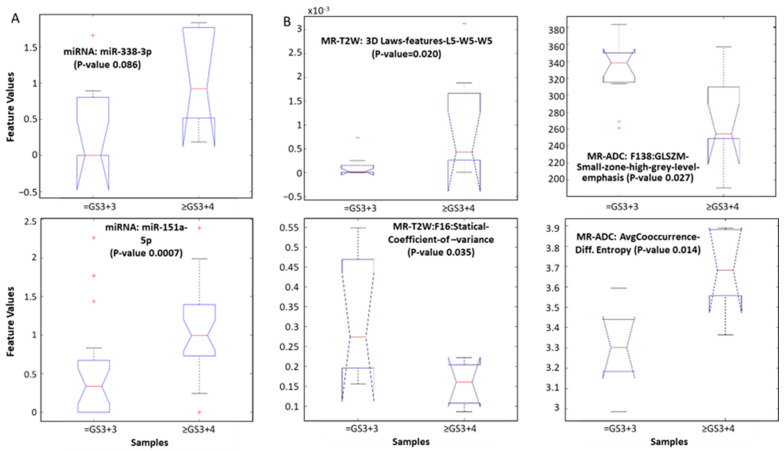
Feature distribution that differentiates clinically significant (≥3 + 4) from indolent (3 + 3) for (**A**) individual cohorts (miRNA, MR T2w, MR ADC), and (**B**) combined cohort (miRNA, MR T2w, MR ADC). Significance computed using non-parametric Wilcoxon rank test.

To identify individual miRNAs and image features that were associated with aggressive PCa, we first performed correlation analysis and removed correlated features (R^2^ ≥ 0.99) across all possible features in a modality (miRNA, *n* = 48; MR-T2W/ADC, *n* = 18 biopsies). This step removed 6.8%, 46.7%, and 40.8% of the metrics, leaving us with 300, 163, and 181 uncorrelated features for miRNA, MR-T2W, and MR-ADC modalities, respectively. While in the matched cohort (imaging and miRNA, *n* = 13), we had 285 (removed 11.4%), 143 (removed 53.2%), and 166 (removed 40.8%) uncorrelated features for miRNA, MR-T2W, MR-ADC modalities, respectively. We then performed non-parametric tests and identified individual features that were statistically significantly different between indolent and clinically significant patients (Table 1 and Figure 2).

**Figure 3:**
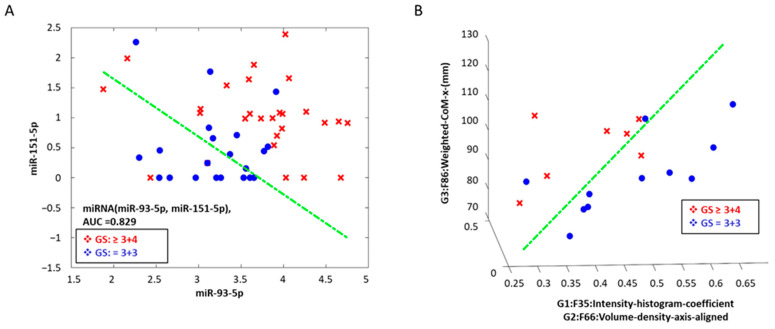
Multi-feature scatter plot shows the spread of aggressive from indolent grade prostate cancer along with the discrimination boundary (**A**) using miRNA-based features (miR-93-5p, miR-151a-5p) and (**B**) MR-T2w features (3-features).

We used clinical variables (age, PSA, and PIRADS) across sub-cohorts to form predictors to discriminate aggressive grades (see Supplementary Table S2). We then built classifier models using logistic regression with univariate and multivariable (2 and 3) features. The predictive ability of these models was assessed based on area under the receiver operator characteristics (AUC) using a cross-validation (hold out) approach. For univariate feature-based models using either miRNA or imaging modalities, we found that the miRNAs (miR-93-5p and miR-151-5p)-based model had an average AUC in the range of 0.66–0.76. MR-T2W radiomic features (Laws-features) had an average AUC range from 0.78 to 0.87, while MR-ADC radiomic features (co-occurrence, volume, wavelet) showed an average AUC range from 0.78 to 0.84 (see Table 2). Figure 3 shows an example of univariate feature-based classifiers.

### 3.3. Multimodal Classifier Model

**Figure 4:**
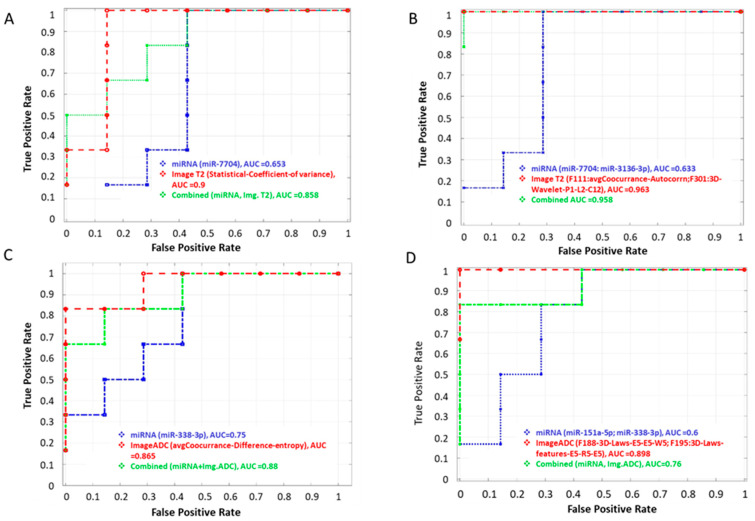
Receiver operating characteristic curve (ROC) in using top predictors to discriminate clinically significant (Gleason 3 + 4) from indolent grade prostate cancers. (**A**) miRNA (miR-7704) with T2W radiomics (univariate), (**B**) miRNA (miR-338-3p) with ADC radiomics (univariate), (**C**) miRNA (miR-7704, miR-3136-3p) with T2W radiomics (2-features), and (**D**) miRNA (151a-5p,338-3p) with ADC radiomics (2-features).

To evaluate if the combination of multimodality features could improve the performance of detecting aggressive disease, we built multimodal predictors by combining miRNAs and bi-parametric MR features (MR-T2W/ADC) from respective modalities in the matched cohort (*n* = 13). In multimodal feature analysis, we selected the best univariate miRNA’s that had functional relevance to prostate oncology. Using miRNA and MR-T2W radiomics, univariate features had an average AUC of 0.65 to 0.71 and 0.77 to 0.90, respectively. Combining these two features (miRNA and MR-T2W radiomics) produced an average AUC of 0.73 to 0.86. Thus, the two feature-based models (miRNA: miR-7704, miR-151a-5p, T2W: COV, Co-Occurrence) combining all modalities produced a more predictive and successful model with an AUC range from 0.79 to 0.96. While using a single feature from miRNA and MR-ADC modalities, a combination (miRNA and MR-ADC) had an average AUC range from 0.73 to 0.75, 0.87 to 0.90, and 0.77 to 0.88, respectively. While using two features from each of the modalities (miRNA: miR-151a-5p, miR-338-3p and MR-ADC: co-occurrence, Laws features) improved AUC (average range from 0.76 to 0.88) in comparison to using them individually. Importantly, the sensitivity/specificity was higher compared to individual modality-based models (see Table 3 and Figure 4).

### 3.4. Gene Ontology and Regulatory Pathways

**Figure 5:**
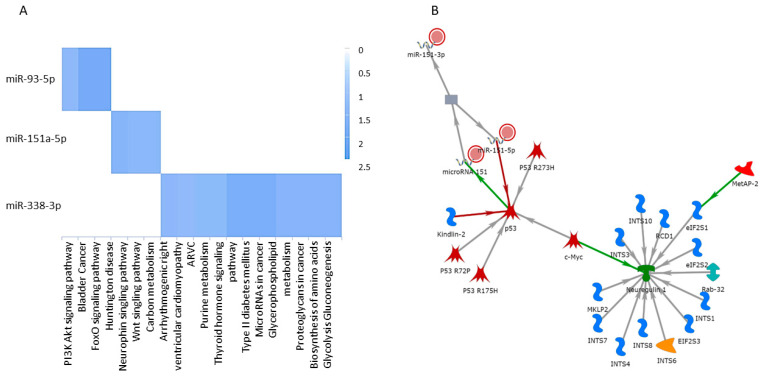
Regulator pathways enriched for miRNAs (miR-151a-5p, miR-338-3p, and miR-93-5p) that were identified in our predictive analysis. Pathway tools using; (**A**) GO functional pathway (Pather.org), (**B**) p52 pathway enriched, seeded with two miRNAs (miR-151a-5p, miR-338-3p) using (GeneGo^®^).

After identifying top miRNAs (miR-151a-5p, miR-338-3p, miR-7704, miR-93-5p, and miR-190b-5p) that were predictors of aggressive PCa, we used these markers to link regulatory pathways associated with their predicted targets using the following curated databases: TargetScan (https://www.targetscan.org/vert_80/ accessed on 10 May 2024) and miRDB target computational prediction software. We also evaluated other open-source pathway miner tools (KEGG [34], PANTHER [35], and Database for Annotation, Visualization, and Integrated Discovery (DAVID) [36]). The data-mining analysis identified the most relevant pathways using miRNAs as seeds that were most common between TargetScan and miRDB. We found a significant enrichment in the following gene-pathway associations: Pathways in cancer, PI3K, Akt signaling, FoxO signaling, and Wnt signaling pathway genes, reported by PANTHER (see Figure 5A). In addition, Ras signaling, angiogenesis, FGF signaling, Wnt signaling, and PDGF (Platelet-derived growth factor) signaling pathways were the most significant pathways identified using the KEGG pathway (see Figure 5B).

## 4. Discussion

PCa diagnosis and treatment strategies have seen improvements in the last two decades [37,38,39,40]. Despite this, early detection of clinically significant cancers remains challenging [40], especially since PSA-based tests have resulted in a high level of false diagnoses [3,41]. Genomic-based technologies are believed to provide a promising tool for identifying aggressive disease [38,42], and reducing the incidence of false positive diagnoses. Clinical use of genomic markers to identify the disease potential of metastasis has improved the management of care for these patients [43]. In previous work, the use of extracellular miRNAs has been shown to improve disease detection across various oncological diseases, including PCa [25,44]. Urine macrovesicle-associated biomarkers hold significant potential as non-invasive tools for cancer detection and prognosis [45]. These biomarkers can reflect tumor-specific molecular signatures, offering insights into disease progression and therapeutic response [46]. Their accessibility and correlation with tumor biology make them valuable for monitoring disease dynamics, particularly in prostate cancer [47]. Further validation of these biomarkers and comparison with other modalities would enhance their clinical utility in personalized medicine approaches. In this pilot study, we assembled a cohort of primary prostate cancer patients (*n* = 48) with matched high-quality prostate MR imaging (*n* = 13), of which some patients had multiple lesions (*n* = 18), through a retrospective analysis.

In this study, we identified discriminant biomarkers using individual modalities (miRNA, MR-T2/ADC) and, in combination, have shown them to be related to clinically significant prostate disease (see Table 2 and Table 3). We formed predictors using clinical variables (PSA, PIRADS-v2) that served as baseline comparators (See Supplementary Table S2).

Similarly, quantitative imaging or radiomic features that describe the physiology of the tumor also describe the pixel-based patterns that include histogram intensity, co-occurrences, textures; some of these metrics are shown to be predictive of aggressive prostate disease, and these metrics have been related to biochemical recurrence [48,49]. Recent work has implicated radiomic metrics related to first-order statistics, texture (Law features, Haralick/co-occurrence) features extracted in MR-T2w, and radiomics features related to texture, edge descriptors (Laws, gradient, Sobel) computed in MR-ADC in identifying aggressive disease grades [49]. In accordance with prior work, our study finds several imaging features related to intensity and texture-based features (co-occurrence, wavelets, Laws) in T2W/ADC modalities that can help identify aggressive prostate disease (see Table 2 and Table 3). Despite the smaller sample size that could provide higher estimates, we tried to mitigate this effect by using cross-validation methods with randomization and reported ensemble results. Our findings in this study show a similar trend to previously reported results [50,51].

We and others have demonstrated that specific miRNAs are differentially expressed in PCa, making them valuable for early diagnosis and disease monitoring [52,53]. Our study, utilizing a univariate feature-based model with miRNA and imaging modalities, identified miR-151a-5p and miR-93-5p as having the highest AUC (see Table 2). We found other promising combinations of miRNA and imaging features, as presented in the Supplemental Section (See Supplementary Table S1). miR-151a-5p is differentially expressed in PCa, indicating its role in tumor aggressiveness [54]. This miRNA is well-known as an oncogene, particularly in colorectal cancer, and it is also overexpressed in lung cancer and lymphoblastic leukemia [55,56,57]. Our KEGG analysis predicted that miR-151a-5p indirectly targets the Neuregulin 1 (NRG1) gene through P53 and c-Myc. Recent studies have also shown that the NRG1 gene promotes antiandrogen resistance in PCa [58]. The consistent dysregulation of miR-151a-5p across various cancers highlights its universal role in oncogenic processes. The overexpression of miR-93-5p has been linked to increased migration and invasion in squamous cell carcinoma of the head and neck, suggesting an oncogenic role [59,60]. While the exact mechanism of action needs further investigation, elevated levels of miR-93-5p have also been associated with epithelial–mesenchymal transition (EMT), radiotherapy response, and poor prognosis [60,61,62]. Additionally, miR-93-5p has been shown to be upregulated in oral cancer [63,64]. These findings reinforce the role of miRNAs in regulating PCa, a foundation for further research into their mechanistic roles and therapeutic potential (See Table 4).

In our multimodal analysis using miRNA and bi-parametric MR features (MR-T2W, MR-ADC), we identified miR-7704, miR-3136-3p, miR-151a-5p, and miR-338-3p as having high AUC values. miR-151a-5p and miR-328-3p were identified as top predictors when combined with radiomics data, underscoring their potential as non-invasive biomarkers for prostate cancer. miR-151a-5p has been reported to act as a proto-oncogene by regulating apoptosis and cell proliferation pathways, particularly in prostate cancer progression [54]. Similarly, miR-328-3p is known to influence tumorigenesis by modulating pathways associated with cell migration and invasion, suggesting its utility in monitoring aggressive prostate cancer [67].

Based on other studies, miR-7704 emerges as a potential target for aggressive PCa, consistent with its reported roles in ovarian and breast cancers [68,69]. In ovarian cancer, miR-7704 is part of a feedback loop with IL2RB and AKT, influencing tumorigenesis and chemoresistance [68]. This suggests that miR-7704 may play a critical role in cancer progression and underscores its potential as a therapeutic target and prognostic biomarker across different cancer types. In another study, miR-3136-3p was significantly upregulated in high-grade cervical intraepithelial neoplasia in liquid biopsy samples [70]. miR-338-3p is downregulated in several cancers, including gastric, ovarian, and breast cancers [71,72,73,74]. In PCa cells, overexpression of the miR-338-3p suppresses cell migration and invasion [75,76,77].These miRNAs exhibited high AUC in our multimodal miRNA and bi-parametric MR feature-based analyses, indicating their strong diagnostic potential for aggressive PCa. They may serve as valuable therapeutic targets and prognostic biomarkers across various cancer types (see Table 3 and Table 4 and Figure 4 and Figure 5).

Although single omics analysis has shown promise in identifying aggressive disease, the PCa is known to be highly heterogeneous. One omics data may not capture the complete landscape of PCa biology [78,79]. It is believed that multi-omic approaches can increase the sensitivity of biomarkers. Therefore, we evaluated the multi-omic approach in a matched sample cohort with pretreatment blood plasma and MRI to identify biomarkers in localized PCa patients. Our data showed that several miRNA and image-based features are differentially expressed in clinically significant PCa. By combining circulating extracellular transcriptomes and MRI-based radiomics, our multi-omic model improved performance in distinguishing aggressive diseases. We demonstrate how the integration of these features significantly enhances the accuracy of predicting clinically significant PCa, demonstrating the value of a mixed-modality approach in assessing disease aggressiveness.

## 5. Limitations

Our study has a relatively small sample size, which may affect the generalizability of the findings. Additionally, reliance on a single institutional cohort may introduce bias and limits. The racial cross-sectional nature of the data could add to biases. We used several mitigation strategies that include cross validation approaches. The study also did not account for potential confounding factors such as providers, patient racial/treatment history, or genetic variability. Further research with larger, diverse cohorts and longitudinal data would validate and expand upon these results.

## 6. Conclusions

Our study findings highlight the significant roles of circulating transcriptomics and radiomics in identifying pathologically aggressive PCa. By outlining specific miRNAs and MRI features, the study enhances our understanding of PCa pathogenesis through improved assessment of morphological characteristics. The combination of prostate miRNAs with imaging metrics offers a non-invasive method for assessing aggressive disease. Our matched cohort’s sample size is limited, but it still provides promising biomarkers. However, validation of these findings in the secondary, independent cohort will ascertain the reported findings.

## Figures and Tables

**Table 1:** Patient cohort characteristics and feature descriptors across modalities.

Modalities	Patient Samples (Gleason Score)	Age (Mean, SD, Median)	PSA (Mean, SD, Median)	Number of Features	Uncorrelated Features (*R*^2^ ≥ 0.99)	Number of Significant Features (Wilcoxon, *p* ≤ 0.05)	
						3 + 3 vs. ≥3 + 4	3 + 3 vs. ≥4 + 3
Individual Modalities							
miRNA	48 (3 + 3: 21, 3 + 4: 14, ≥3: 13)	75, 7.25, 76	7.09, 11.9, 5.14	322	300	27	28
Radiomics on MRI: T2W	18 (3 + 3: 11, 3 + 4: 5, ≥4 + 3: 2)	72.7, 7.1, 75	7.1, 5.76, 6	306	163	13	N/A
Radiomics on MRI: ADC				306	181	5	N/A
Matched Samples (across modalities)							
miRNA	13 (3 + 3: 7 3 + 4: 4 ≥4 + 3: 2)	72.7, 7.1, 75	7.1, 5.76, 6	322	285	5	N/A
Radiomics on MRI: T2W				306	143	4	N/A
Radiomics on MRI: ADC				306	166	3	N/A

**Table 2:** Features that discriminate clinically significant prostate cancer from indolent (3 + 3) using independent features in these modalities; miRNA—univariate (GS 3 + 3 vs. ≥3 + 4, *n* = 48), miRNA—univariate (GS 3 + 3 vs. ≥4 + 3, *n* = 34), univariate imaging T2W (GS 3 + 3 vs. ≥3 + 4, *n* = 18), and univariate imaging ADC (GS 3 + 3 vs. ≥3 + 4, *n* = 18).

Serial No.	miRNA (1-Marker)	Sensitivity/Specificity	PPV/NPV	AUC [CI], SD
miRNA—Univariate (GS 3 + 3 vs. ≥3 + 4, *n* = 48)				
1.	miR-151a-5p	0.798/0.68	0.756/0.726	0.76 [0.388–0.956], 0.162
2.	miR-338-3p	0.807/0.573	0.696/0.709	0.699 [0.213–0.956], 0.201
3.	miR-93-5p	0.775/0.547	0.692/0.67	0.767 [0.457–0.971], 0.15
4.	miR-208a-5p	0.833/0.482	0.671/0.686	0.658 [0.2–0.95], 0.194
5.	miR-190a-5p	0.842/0.469	0.654/0.687	0.702 [0.392–0.968], 0.173
miRNA—Univariate (GS 3 + 3 vs. ≥4 + 3, *n* = 34)				
1.	miR-93-5p	0.684/0.857	0.762/0.798	0.821 [0.3–0.95], 0.208
2.	miR-8072	0.631/0.855	0.74/0.767	0.776 [0.05–0.95], 0.237
3.	miR-328-3p	0.589/0.861	0.727/0.73	0.798 [0.417–0.958], 0.178
4.	miR-1469	0.574/0.845	0.652/0.772	0.772 [0.333–0.958], 0.183
5.	miR-5583-5p	0.539/0.821	0.645/0.728	0.786 [0.25–0.958], 0.202
Univariate Imaging T2W (GS 3 + 3 vs. ≥3 + 4, *n* = 18)				
1.	3D_Laws_features_L5_E5_S5	0.588/0.882	0.627/0.748	0.83 [0.05–0.95], 0.267
2.	3D_Laws_features_L5_E5_W5	0.522/0.897	0.645/0.713	0.871 [0.325–0.963], 0.192
3.	3D_Laws_features_L5_S5_S5	0.575/0.802	0.587/0.712	0.827 [0.05–0.95], 0.264
4.	3D_Laws_features_L5_W5_W5	0.432/0.925	0.565/0.688	0.853 [0.2–0.95], 0.238
5.	3D_Laws_features_E5_E5_L5	0.448/0.892	0.557/0.699	0.778 [0.05–0.95], 0.312
Univariate Imaging ADC (GS 3 + 3 vs. ≥3 + 4, *n* = 18)				
1.	Volume_density_minimum_volume_enclosing_ellipsoid	0.72/0.832	0.702/0.808	0.844 [0.367–0.967], 0.208
2.	Maximum_histogram_gradient_grey_level	0.413/1	0.61/0.708	0.777 [0.525–0.975], 0.174
3.	GLSZM_Small_zone_high_grey_level_emphasis	0.582/0.81	0.663/0.693	0.818 [0.3–0.95], 0.234
4.	avgCoocurrence_Difference_entropy	0.605/0.758	0.546/0.759	0.801 [0.05–0.95], 0.263
5.	3D_Wavelet_P1_L2_C12	0.648/0.71	0.576/0.714	0.817 [0.325–0.963], 0.22

**Table 3:** Multimodal features based (miRNA and imaging) model to discriminate clinically significant prostate cancer (≥3 + 4) from indolent (3 + 3) in a matched cohort of patients (*n* = 13). (A,B) extracellular exosomal miRNA with MRI T2W. (C,D) extracellular exosomal miRNA with MRI ADC features.

Serial No.	miRNA	Imaging	AUC [CI], SD		
			miRNA	Image: T2W	Combined (miRNA, T2W, ADC)
(A) Combined: miRNA + Image (T2W): (GS 3 + 3 vs. ≥3 + 4): 1-dimension					
1.	miR-151a-5p	Avg_Coocurrence_Joint_MAX	0.713 [0.05–0.95], 0.347	0.77 [0.05–0.95], 0.358	0.73 [0.05–0.95], 0.344
2.	miR-7704	Statistical_Coefficient_of_variance	0.653 [0.05, 0.95], 0.371	0.9 [0.4–0.95], 0.225	0.858 [0.4–0.95], 0.223
(B) Combined: miRNA + Image (T2W): (GS 3 + 3 vs. ≥3 + 4): 2-dimension					
1.	miR-151a-5p, miR-6717-5p	Volume_at_intensity_fraction_10, Weighted_CoM_x_(mm);	0.978 [0.7, 0.95], 0.118	0.948 [0.525–0.975], 0.134	0.95 [0.2–0.95], 0.178
2.	miR-7704, miR-3136-3p	Avg_Coocurrence_Autocorrelation, 3D_Wavelet_P1_L2_C12;	0.633 [0.05, 0.95], 0.368	0.963 [0.525–0.975], 0.12	0.958 [0.525–0.975], 0.128
3.	miR-151a-5p, miR-338-3p	GLSZM_High_grey_level_zone_emphasis, 3D_Wavelet_P1_L2_C12;	0.573 [0.05, 0.95], 0.406	0.898 [0.05–0.95], 0.219	0.785 [0.4–0.95], 0.238
(C) Combined: miRNA + Image (ADC): (GS 3 + 3 vs. ≥3 + 4): 1-dimension					
1.	miR-338-3p	Avg_Coocurrence_Difference_entropy	0.75 [0.05, 0.95], 0.314	0.865 [0.05–0.95], 0.29	0.88 [0.475–0.963], 0.212
2.	miR-151a-5p	Volume_density_minimum_volume_enclosing_ellipsoid	0.733 [0.05, 0.95], 0.318	0.895 [0.2–0.95], 0.241	0.768 [0.05–0.95], 0.312
(D) Combined: miRNA + Img (ADC): (GS 3 + 3 vs. ≥3 + 4): 2-dimension					
1.	miR-190b-5p; miR-106b-3p	Avg_Coocurrence_Difference_entropy, avg_3D_LGRE_(Low_grey_level_run_emphasis)	0.843 [0.05, 0.95], 0.272	0.88 [0.05–0.95], 0.276	0.88 [0.4–0.95], 0.237
2.	miR-151a-5p; miR-338-3p	3D_Laws_features_E5_E5_W5, 3D_Laws_features_E5_R5_E5	0.6 [0.05, 0.95], 0.381	0.898 [0.05–0.95], 0.256	0.76 [0.2–0.95], 0.261

**Table 4:** Predictive biomarker and their relationship to the biological pathways (HOXA: homeobox A cluster, TGF-β1: transforming growth factor beta1, Smad3: Mothers against decapentaplegic homolog 3, PHLPP1: PH Domain And Leucine Rich Repeat Protein Phosphatase).

Pathways			
	Biomarkers	Target Gene	Biological Function
miRNA’s			
1	miR-151a-5p, miR-338-3p	Neuregulin 1 (HOXA, p53)	Promotes proliferation and metastasis. Inhibits proliferation and promotes apoptosis
2	miR-93-5p miR-208a-5p miR-190a-5p	TGF-β1/Smad3 N/A PHLPP1	miR-93-5p targets Smad7 to regulate the transforming growth factor-β1/Smad3 pathway [65] N/A Promoting migration and invasion [66]
Radiomics (MRI)			
1	T2W: Volume_at_intensity_fraction; Weighted_CoM; Avg_Coocurrence_Autocorrelation; 3D_Wavelet_P1_L2_C12; GLSZM_High_grey_level_zone_emphasis.	Texture, morphology related to heterogeneity	Disease heterogeneity, proliferation
2	AvgCoocurrence-Difference_entropy; Volume_density_minimum; Volume_enclosing_ellipsoid	Texture, shape, density	Disease proliferation

TGF-β1: Transforming growth factor beta-1 (TGF-β1), Smad3: Mothers against decapentaplegic homolog 3, PHLPP1: PH Domain And Leucine Rich Repeat Protein Phosphatase 1.

## Data Availability

All data produced in the present study will be shared after the institutional data transfer agreement.

## Supplementary Materials

The following supporting information can be downloaded at: https://www.mdpi.com/article/10.3390/cancers17050835/s1, Table S1: Univariate miRNA predictors; Table S2: Clinical variables ability to discriminate clinically significant prostate cancer from indolent (3 + 3) in below sub-cohorts; miRNA, ≥3 + 4 (*n* = 48), miRNA, ≥4 + 3 (*n* = 34), MRI (T2w/ADC) (*n* = 18) and Combined miRNA and MRI (*n* = 13); Table S3: Quantitative Image features used to describe the lesion of interest, computed independently in each imaging modality [32,80]; Table S4: Description of Texture Features [81,82,83,84,85,86,87,88].

## Abbreviations

PCa: Prostate cancer, PSA: Prostate-Specific Antigen, miRNAs: microRNAs, PBS: Phosphate Buffered Saline, ROC: Receiver Operating Characteristic, MRI: Magnetic Resonance Imaging, RP: Radical Prostatectomy, BCR: Biochemical Recurrence, PIRADS: Prostate Imaging Reporting and Data System, EDTA: Ethylenediaminetetraacetic Acid, T2W: T2-weighted, DWI: Diffusion-Weighted Imaging, GS: Gleason Score, ADC: Apparent Diffusion Coefficient, IBSI: Image Biomarkers Standardization Initiative, SD: Standard Deviation, *p*: *p*-Value, R^2^: Coefficient of Determination, T2W: T2-weighted, ADC: Apparent Diffusion Coefficient. PPV: Positive Predictive Values, NPV: Negative Predictive Values, AUC: Area Under the Receiver Operator Curve, CI: Confidence Interval, SD: Standard Deviation. CI: Confidence Interval.
